# Etiology, Clinical Profile, and Short-Term Outcome of Children With Acute Kidney Injury

**DOI:** 10.7759/cureus.22563

**Published:** 2022-02-24

**Authors:** Shabeeta Bai, Khemchand N Moorani, Bilquis Naeem, Muhammad Ashfaq, Rajesh ., Ejaz Ur Rehman

**Affiliations:** 1 Pediatric Nephrology, National Institute of Child Health, Karachi, PAK; 2 Pediatrics, National Institute of Child Health, Karachi, PAK; 3 Medicine, Sir Syed College of Medical Sciences for Girls, Karachi, PAK; 4 Pediatrics, United Medical and Dental College, Karachi, PAK

**Keywords:** end-stage renal disease, outcome, clinical profile, etiology, acute kidney injury

## Abstract

Background: Acute kidney injury (AKI) is a common clinical syndrome in hospitalized children and it imposes heavy burden of mortality and morbidity. In resource-constraint settings, management of AKI is very challenging and associated with adverse outcomes. The aim of this study was to determine the clinico-etiological profile and outcome of AKI.

Methodology: This prospective observational study was done at the department of pediatric nephrology and pediatric intensive care unit, National Institute of Child Health, Karachi, Pakistan from December 2020 to May 2021. A total of 130 children aged 1 month to 15 years, diagnosed with AKI irrespective of the underlying cause were included. Detailed medical information of each child including medical history, examination, and baseline investigations were obtained. Clinical and etiological profile of patients was noted. The patients were followed up to three months and the outcome was noted.

Results: In a total of 130 children, 82 (63.1%) were male. The mean age was 5.5±4.4 years (ranging between 1 month and 15 years). There were 117 (90.0%) children who were referred from other centers for either dialysis or surgical treatment. Prerenal cause of AKI was found in 66 (50.8%) children, followed by renal 53 (40.8%) and postrenal in 11 (8.5%) cases. Fever and shortness of breath were the most common clinical presenting symptoms in 102 (78.5%) and 100 (76%) cases, respectively. There were 45 (34.6%) cases who were managed conservatively, 80 (61.5%) needed dialysis, while three children were managed with plasmapheresis and two required surgical intervention in the emergency department. At three-month follow-up period, 64 (49.2%) children recovered (including nine with partial recovery), 46 (36.1%) expired, 9 (6.9%) developed end-stage renal disease, while 11 (8.5%) had chronic kidney disease.

Conclusion: Sepsis, nephrotoxic drugs, and acute glomerulonephritis were the major causes of AKI at our center. Mortality was high among children presenting with AKI. A relatively high proportion of children with younger age, septic AKI, and presentation in critical condition could be the reasons for this high mortality.

## Introduction

Acute kidney injury (AKI) is a common clinical syndrome in hospitalized children and it imposes heavy burden of mortality and morbidity. More than 13 million people suffer from AKI each year and 85% of them live in developing countries [1]. Further, it is estimated that 1.7 million deaths occur each year from AKI and 60% of AKI survivors may experience residual renal abnormalities: proteinuria, hypertension, and reduced glomerular filtration rate (GFR) [2]. Incidence, etiology, and outcome of pediatric AKI are quite variable and depend upon age, geographic region, and clinical setting. A prospective study from India reported the prevalence of AKI in critically ill patients as 6% while development was independently linked with a significantly higher 30-day mortality rate [3]. Global data report mortality rates between 11% and 63% among children with AKI [4,5]. A study from Pakistan reported oliguria/anuria (83.6%), hypertension (37.1%), and severe anemia (17.2%) to be the most common presentations among children with AKI while post-infectious glomerulonephritis (PIGN) and crescentic glomerulonephritis in primary renal, obstructive urolithiasis in postrenal, and sepsis in prerenal were the most common etiologies [6].

Differences exist regarding the most common presentation and etiologies among children with AKI, and it is necessary to analyze the presentation, etiology, and outcome at our institute. National Institute of Child Health (NICH) is one of the largest public sector hospitals in Karachi, Pakistan, where diagnostic facilities and management are provided free of cost. Neonatal intensive care unit and pediatric ICU (PICU) facilities are available, so the young children with AKI who need peritoneal dialysis are mostly referred to our center. This study was designed to prospectively determine the etiology, clinical profile, and treatment outcome of AKI in children.

## Materials and methods

Design, place, and duration of the study

This is a prospective observational study. This study was conducted at the department of pediatric nephrology and PICU, NICH, Karachi, Pakistan from December 2020 to May 2021.

Inclusion and exclusion criteria

Children aged one month to 15 years, diagnosed with AKI irrespective of the underlying cause were included. Known cases of chronic kidney disease (CKD) or those with congenital anomalies of kidneys and urinary tract were excluded.

Data collection

Institutional Ethical Review Board (IERB) of NICH Karachi approved this study (IERB No: 40/2020). Informed and written consents were acquired from parents/guardians/caregivers of all study participants. Detailed medical information of each child including medical history, examination, and baseline investigations, i.e. complete blood count, urea, creatinine, electrolytes, urinalysis, urine, and blood cultures, and ultrasound of kidneys, ureters, and bladder were obtained. The diagnosis of AKI was based on pRIFLE (Pediatric Risk, Injury, Failure, Loss, End Stage Renal Disease) criteria [7]. Estimated GFR was determined by using Schwartz equation [8]. Further workup was done according to need in individual case like complement 3, antinuclear antibody, anti-dsDNA, and renal biopsy. All children with AKI were managed as per institutional protocol. Indications for dialysis were one or more of the following: complete anuria for >24 hours, volume overload, refractory metabolic acidosis, advanced uremia or uremic encephalopathy, and severe electrolyte imbalance. Peritoneal dialysis was the preferred modality in children <10 kg and children with unstable hemodynamics. The patients were monitored daily with serum creatinine and urine output before, during dialysis, and till discharge. The patients were followed up to three months according to clinical status at discharge and laboratory parameters.

For each patient, predesigned proforma was filled recording to relevant details. Severity of AKI at presentation was graded using pRIFLE criteria. Etiology of AKI was grouped as prerenal, renal, and postrenal. Outcome of AKI was measured in terms of mortality, partial recovery (CKD, proteinuria, hypertension, recurrent urinary tract infection, normalization of urinary stream), and need for long-term dialysis to label as end-stage renal disease (ESRD).

Statistical analysis

SPSS version 26.0 (IBM Corp, Armonk, NY) was employed for data analysis. Qualitative data were expressed as frequency and percentages. Quantitative variables were represented as mean and standard deviation. Chi-square test was performed comparing the outcome of children as per pRIFLE criteria considering P <0.05 as significant.

## Results

In a total of 130 children, 82 (63.1%) were male and 48 (36.9%) were female with a male-to-female ratio of 1.7:1. The mean age was 5.5±4.4 years (ranging between one month and 15 years). The most common age group was between 5 and 15 years accounting for 46.9% cases followed by 1-5 years of age (29.2%) and infants (23.8%). Majority of the patients, 78 (60.0%), belonged to urban areas. Poor socioeconomic status was noted in 78 (60.0%) children. There were 117 (90.0%) children who were referred from other centers for either dialysis or surgical treatment.

Etiology of AKI

Prerenal cause of AKI was found in 66 (50.8%) children, followed by renal 53 (40.8%) and postrenal in 11 (8.5%) cases. The most common etiology in prerenal and renal AKI was infectious followed by drug-induced. Among the infectious etiologies of AKI, urosepsis was the most common, 24 (36.4%), followed by acute gastroenteritis (AGE) in 15 (22.7%), malaria in 5 (9.4%), tetanus in 5 (7.8%), dengue shock syndrome in 2 (3.0%), meningococcemia in 1 (1.5%), and diphtheria in 1 (1.5%). Hepatorenal syndrome secondary to acute viral hepatitis was observed in 2 (3.0%) cases. Sepsis and AGE were the most common causes of AKI in children <5 years. Among the intrinsic category of AKI, drug-induced AKI and hemolytic uremic syndrome were observed in 16 (30.2%) and 13 (24.5%), respectively. In drug-induced AKI, vancomycin was found in eight cases while captopril was found in five cases. Among the postrenal etiologies, obstructive urolithiasis was observed in nine (81.8%) cases and two (18.2%) had pelvi-ureteric junction obstruction. Table 1 shows the etiologies of AKI.

**Table 1:** Etiology of acute kidney injuries in children

Etiologies of Acute Kidney Injury		Number	(%)
Prerenal (n=66)	Sepsis	24	36.4
	Acute gastroenteritis	15	22.7
	Diabetic ketoacidosis	8	12.1
	Tetanus	5	7.8
	Meningococcemia	1	1.5
	Diphtheria	1	1.5
	Dengue shock syndrome	2	3.0
	Hepatorenal syndrome	2	3.0
	Congenital adrenal hyperplasia	1	1.5
	Tumor lysis syndrome	4	6.1
	Myocarditis	3	4.5
Renal causes (n=53)	Drug-induced	16	30.2
	Post-infectious glomerulonephritis	6	11.3
	Acute tubular necrosis	2	3.8
	Membranoproliferative glomerulonephritis	2	3.8
	Lupus nephritis	2	3.8
	Crescentic glomerulonephritis	2	3.8
	Rapidly progressive glomerulonephritis	2	3.8
	Hemolytic uremic syndrome	13	24.5
	Tubulointerstitial nephritis	1	1.9
	Malarial nephropathy	5	9.4
	Scorpion bite	2	3.8
Postrenal (n=11)	Stone	9	81.8
	Pelvi-ureteric junction obstruction	2	18.2

Fever and shortness of breath were the most common clinical presenting symptoms in 102 (78.5%) and 100 (76%) cases, respectively. Oliguria/anuria was noted in 69 (53.1%) and altered sensorium in 67 (51.5%) were also found. There were 46 (35.4%) children who needed inotropic support. On examination, 54 (41.5%) had signs of fluid overload and 60 (46.2%) had signs of shock, while 24 (18.5%) were hypertensive. Hypernatremia was noted in 46 (35.4%) children. Table 2 shows the clinical profile of children with AKI.

**Table 2:** Clinical and laboratory features of children with acute kidney injury

Clinical Profile	n	%
Fever	102	78.5
Shortness of breath	100	76.9
Oliguria/anuria	69	53.1
Altered sensorium	67	51.5
Loose motion	43	33.1
Vomiting	62	47.7
Sign of fluid overload	54	41.5
Inotropic support	46	35.4
Signs of shock	60	46.2
Blood Pressure >95th centile	24	18.5
Anemia	68	52.3
Dehydration	24	18.5
Rashes	9	6.9
Leucocyte count >15,000	63	47.7
Thrombocytopenia	40	30.8
Hypernatremia	46	35.5
Echogenic kidneys on ultrasonography	27	20.7

Severity of AKI at presentation

As per pRIFLE criteria, the majority of children were in failure category, 119 (91.5%), at presentation, 9 (6.9%) were in injury, and 2 (1.5%) in risk category.

Outcome of children with AKI

There were 45 (34.6%) cases who were managed conservatively, 80 (61.5%) needed dialysis (44 needed peritoneal dialysis and 36 hemodialysis), while three children managed with plasmapheresis and two required surgical intervention in the emergency department. At three-month follow-up period, 64 (49.2%) children recovered (including nine with partial recovery), 46 (36.1%) expired, 9 (6.9%) developed ESRD, while 11 (8.5%) had CKD. Table 3 shows the details of outcome according to pRIFLE criteria at three-month follow-up.

**Table 3:** Outcome according to pRIFLE criteria at three-month follow-up CKD: chronic kidney disease; ESRD: end-stage renal disease; pRIFLE: Pediatric Risk, Injury, Failure, Loss, End-Stage Renal Disease

pRIFLE Categories	Recovered	CKD	ESRD	Death	P-Value
Risk(n=2)	1	-	-	1	0.6247
Injury (n=9)	7	1	-	1	
Failure (n=119)	56	10	9	44	

## Discussion

AKI contributes to high childhood morbidity and mortality and increases significant economic burden on the family and healthcare system [9]. Early identification of risk factors can prevent the initiation of disease and enhance prompt diagnosis and treatment [10]. There is very limited local data regarding risk factors and outcome of AKI especially in children <5 years. AKI in the pediatric age group is associated with various etiologies. Etiology and epidemiology of AKI may vary from center to center within a same country. In our cohort of 130 children with AKI, 63% were male, resulting in a male-to-female ratio of 1.7:1, which is quite similar to the ratio of other developing countries [11,12]. In this study, a majority (91.4%) of the children were in failure category at the time of presentation with GFR <35 mL/min/1.73m^2^ as per pRIFLE criteria. This presentation with severe disease has been reported from low-income countries [12]. Possible reasons could be lack of early diagnosis, late referral, and financial constraint, which is the major concern in Pakistan where majority of people are poor and come to hospital when the child has developed severe disease.

After reviewing literature on AKI we found wide variation in etiology between developed and developing countries. In developing countries community-acquired AKI is the common cause, whereas it is hospital-acquired AKI in developed countries. This study found that the prerenal etiology of AKI was noted in 50.8%, renal in 40.8%, and postrenal in 8.5%. A prospective study from China reported prerenal causes of AKI among 27.7% cases of AKI [13]. Researchers have reported that the most common cause of AKI in children in developing countries are infections [14]. We reported sepsis and AGE to be the predominant etiologies behind AKI. Data from developed countries have labeled sepsis and AGE as the most common etiologies of AKI [12,15]. This could be due to poor hygiene and lack of sanitation.

A study from Nigeria reported that 75.8% of children had stage 3 AKI at the time of hospitalization. Sepsis (41.8%) and primary kidney disease (29.7%) were the most common causes [16]. In a study published in 2016 from Karachi, the results were different from our study where PIGN and urolithiasis were the most common etiologies behind AKI [6]. Exposure to nephrotoxic drugs has been recognized as a risk factor for pediatric AKI [17]. In our cohort, exposure to nephrotoxic drugs was the most common renal cause of AKI. Notably, vancomycin and captopril were the most important agents.

The outcome of AKI is dependent upon factors like age, etiology, and the time the child presented to the healthcare facility [18]. Studies from India have reported higher rates of mortality among children with AKI ranging from 28.5% to 46.3%, whereas researchers from sub-Saharan Africa reported high rates of mortality as well (34%) [19,20]. In our study, mortality rate was high (35.4%) comparatively similar to that reported from other developing countries. A relatively high proportion of children with younger age, septic AKI, and presentation in critical condition could be the reasons for this high mortality.

Limitations

First, data in this study is from a single pediatric nephrology center; therefore, it does not truly reflect the etiological spectrum of pediatric AKI prevalent throughout the country. Secondly, neonates were excluded from this study since their susceptibility and etiology of AKI are considerably different from older infants and children. Post-cardiac surgery children were not included in this study. Nevertheless, this prospective study describes very relevant information on pediatric AKI spectrum encountered at a large tertiary care referral center in a developing country.

## Conclusions

Sepsis, nephrotoxic drugs, and acute glomerulonephritis were the major causes of AKI at our center. Mortality was high among children presenting with AKI. A relatively high proportion of children with younger age, septic AKI, and presentation in critical condition could be the reasons for this high mortality.
